# A case report of severe drug-induced immune hemolytic anemia caused by piperacillin

**DOI:** 10.3389/fimmu.2024.1478545

**Published:** 2024-11-06

**Authors:** Hong Zhao, Jian Chen, Guojin Ou

**Affiliations:** ^1^ Department of Laboratory Medicine, West China Second University Hospital, Sichuan University, Chengdu, China; ^2^ Key Laboratory of Birth Defects and Related Diseases of Women and Children Sichuan University, Ministry of Education, Chengdu, China; ^3^ Department of Laboratory Medicine, High-Tech Zone Hospital for Women and Children, West China Second University Hospital, Sichuan University, Chengdu, China

**Keywords:** piperacillin, drug-induced immune hemolytic anemia, autoimmune hemolytic anemia, human leukocyte antigen, transfusion

## Abstract

Piperacillin is a beta-lactamase inhibitor frequently used in the treatment of urinary tract infections. It is a broad-spectrum antibiotic with strong antibacterial action against *Pseudomonas aeruginosa* and *Enterobacter*, especially extended-spectrum beta-lactamase-producing Enterobacteria and *Enterococcus*. Side effects of piperacillin include allergic reactions, rashes such as urticaria, leukopenia, interstitial nephritis, asthma attacks, serological reactions, candida infection, and bleeding with more severe reactions resulting in anaphylactic shock. Anemia and hemolytic anemia are rare adverse reactions to piperacillin, with an incidence of 0.01–0.10%. We report herein the case of a severe postoperative immune hemolytic reaction to piperacillin. Fortunately, we quickly recognized and identified the drug reaction caused by piperacillin, immediately stopped the use of piperacillin, and performed a blood transfusion. The patient recovered and was subsequently discharged from the hospital.

## Introduction

Piperacillin is the third most common antibiotic to induce hemolytic anemia, after cefotetan and ceftriaxone. Studies have shown that patients treated with piperacillin should be evaluated for drug-induced immune hemolytic anemia (DIIHA) if they develop new anemia or an increase in anemia (1). DIIHA is a secondary form of autoimmune hemolytic anemia (AIHA), which is characterized by the increased destruction of red blood cells (RBCs), triggered by autoantibodies that target antigens on the surface of RBCs (2, 3). DIIHA accounts for approximately 10% of all AIHA cases, with an estimated incidence of 1–3 cases per 100,000 people per year (4). The pathogenesis of AIHA is multi-factorial, including genetic factors, infection, autoimmune diseases, and drugs. DIIHA, however, is rare and difficult to diagnose, with knowledge and clinical experience forming the basis for identifying this phenomenon. Diagnoses are typically obtained by combining a patient’s clinical history with the presentation of hemolytic anemia, relying primarily on the identification of anti-RBC autoantibodies through a direct antiglobulin test (DAT) followed by the exclusion of other potential causes of hemolytic anemia. Mayer et al. (5) reviewed 73 cases of DIIHA from 1996–2005 in a German institute, 13 of which were the result of piperacillin administration. All 13 patients presented with acute hemolysis and had positive DAT results.

We report herein the case of a 27-year-old woman who experienced severe hemolytic anemia due to the use of piperacillin-sulbactam sodium after surgery for endometrial malignancy. Piperacillin-dependent antibodies were detected in her serum; therefore, we ruled out the possibility of isoantibodies. We performed human leukocyte antigen gene complex (HLA) class II antigen typing and found that HLA-DQB1*03-DRB1-09*01 haplotype may potentially mediate DIIHA. This study was approved by the Ethics Committee of the West China Second University Hospital, Sichuan University (2020051).

## Case description

A 27-year-old woman was diagnosed with highly differentiated endometrial adenocarcinoma (stage IVc) and underwent surgical treatment 7 years ago. Chemotherapy was administered after the surgery, while platinum drug allergies was discovered during the chemotherapy. Admitted to the hospital due to the discovery of a pelvic and abdominal mass for over 7 months, she was conducted laparotomy for endometrial carcinoma, intestinal resection, intestinal anastomosis, enterostomy, abdominal aorta repair, and left common iliac artery repair under general anesthesia. The intraoperative blood loss was 1700 mL. The patient received a type A, RhD-positive, intraoperative transfusion of 3 units (U) of red leukocyte-depleted suspension containing RBCs and 400 mL of frozen fresh plasma. Postoperatively, low-molecular-weight heparin was used for preventive anticoagulation, and cefuroxime was used to prevent infection. The patient’s postoperative Hb was 86 g/dL, she had no obvious bleeding, and routine piperacillin was administered to prevent infection. On the 5^th^ day after surgery, her Hb decreased to 58 g/dL, antibody screening was positive, I(1+), II(1+), III(1+w), DAT (3+) – lactic dehydrogenase (LDH) was 612 IU/L, and unconjugated bilirubin (UCB) was 23.9 uM/L. Therefore, the patient was suspected to have AIHA. Additional clinical information of the patients is presented in **Table 1**. Her Hb increased to 75 g/dL after a transfusion of 3 U RBCs, indicating effective transfusion.

**Table 1:** Summary of the patient’s clinical information.

Days	Laboratory Results						Symptoms and Signs	Medication Treatment	Transfused RBC (U)	Transfusion Reaction (Y/N)
	Hb (g/L)	PLT (*10^9^/L)	WBC (%)	WBC (*10^9^/L)	RET (*10^12^/L)	RET (%)				
0	107	132	64.9	4.3	/	/	Laparotomy for endometrial cancer after chemotherapy	/	3	N
1	/	/	/	/	/	/	Norm	Cefuroxime	/	/
2	86	74	85.9	7.4	/	/	Norm	Piperacillin-tazobactam	/	/
3	88	69	5.9	69.8	/	/	Body temperature increased, with a top of 38°C.	Piperacillin-tazobactam	/	/
4	/	/	/	/	/	/	Norm	Piperacillin-tazobactam	/	/
5	62	60	55.7	3.3	/	/	Norm	Piperacillin-tazobactam	/	/
6	58	53	49.3	2.9	/	/	Norm; Anemic appearance.	Piperacillin-tazobactam	3	Y
7	75	55	61.0	3.8	/	/	Norm; Anemic appearance.	Stop antibiotics	/	/
8	68	50	58.3	3.1	/	/	Norm; Anemic appearance; Fever at night, with a top of 38.5°C.	Stop antibiotics	/	/
9	57	53	55.2	3.4	/	/	Norm; Anemic appearance.	Piperacillin-tazobactam	1.5	Y
10	56	48	76.4	2.9	/	/	Norm; Dizziness and fatigue. Palpebral conjunctiva and nail bed pale, severe anemia appearance.	Piperacillin-tazobactam	3	Y
11	64	56	70.8	4.0	/	/	Norm; Temperature fluctuates between 36.8°C-38°C, poor spirit and sleep, and average appetite. Anemia appearance.	Piperacillin-tazobactam	/	/
12	54	51	54.0	3.1	/	/	Norm; Anemia appearance.	Cefoperazone sodium and sulbactam sodium and Prednisone	/	/
13	/	/	/	/	/	/	Norm; Slightly dizzy; Anemia appearance.	Meropenem and Prednisone	/	/
14	42	56	67.1	4.0	0.0020	0.15	Norm; Dizziness and fatigue, and occasionally flustered. Severe anemia, jaundice.	Meropenem and Prednisone	3	N
15	56	49	76.9	6.7	0.006	0.33	Norm; Dizziness eased.	Meropenem, Prednisone and **IVIG**	/	/
16	55	45	72.7	7.8	0.0034	0.20	Norm; Dizziness eased.	Meropenem, Prednisone and IVIG	/	/
17	52	52	82.0	6.8	0.0094	0.60	Norm; Slightly tired and weak after the activity; Anemia appearance.	Meropenem, Prednisone and IVIG	/	/
18	52	58	71.0	6.8	0.0891	5.57	Norm; Anemia appearance.	Meropenem, Prednisone and IVIG	/	/
19	/	/	/	/	/	/	Norm; Anemia appearance.	Prednisone	/	/
20	93	66	76.5	6.4	/	/	Norm; Anemia appearance.	Prednisone	3*	N
21	/	/	/	/	/	/	Norm; Discharge.	Prednisone	/	/

1. The numbers in “Days” column indicate the day after the operation, the number “0 “ indicates the day on operation.

2. The “/” indicates not involved. The “Y” indicates occurred transfusion reaction, while “N” indicates not occurred.

3. The “Norm” in “Symptoms and Signs” column indicates Vital signs are stable, and general conditions are good.

4. The “IVIG” indicates Intravenous immunoglobulin.

5. The “*” indicates the antigen of the transfused RBC is Jka(-) and Leb(-).

6. The medication dosages: Piperacillin-tazobactam, 4.5g q8h; Piperacillin-tazobactam, 4.5g q8h; Meropenem, 0.5g q8h; Prednisone, 25mg bid; Intravenous immunoglobulin, 0.4g/kg.d.

**Figure 1:**
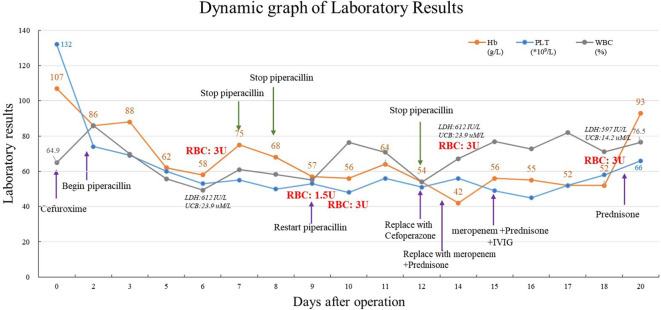
Dynamic graph of laboratory results.

The patient’s Hb dropped to 57 g/dL again on the 9^th^ day after surgery, at which time it was suspected that the autoantibodies had combined with isoantibodies. Her LDH was 915 IU/L, UCB was 21.2 uM/L. The patient experienced fever and chills during the transfusion of 1.5U Jka(-)/Leb(-) RBCs, with the body temperature rose to 39°C half an hour later. Her Hb remained at 56 g/dL, indicating that the blood transfusion had been ineffective. We then evaluated for the presence of drug antibodies, and piperacillin drug antibodies were detected. Piperacillin was discontinued 14 days after surgery, at which time Hb was 42 g/dL. The patient received another 3 U RBC transfusion, as well as simultaneous intravenous immunoglobulin (IVIG) and prednisone therapy. Her Hb increased to 56 g/dL, and the IVIG and prednisone levels were sustained. indicating that the blood transfusion was effective. Her Hb remained at 52 g/dL for 4 days without further change. Another 3U RBC transfusion was administered 18 days after surgery, and her Hb increased from 52 to 93 g/dL, and LDH decreased to 597 IU/L, UCB increased to 14.2 uM/L. Her Hb was re-evaluated 20 days post-discharge, when it was found to be 111 g/dL, and antibody screening was negative. The overall changes in Hb, PLT, and WBC counts are shown in **Figure 1**. Details of serological information are shown in **Table 2**. The patient provided written informed consent before enrolment. This study was approved by the Ethics Committee of the West China Second University Hospital, Sichuan University (2020051).

**Table 2:** Transfusion and immunology related information.

Days	Transfusion and Serological Results						Situations
	Transfused RBC (U)	Antibody Screen	DAT	Major Cross	Minor Cross	Transfusion Reaction and Treatment	
0	3	Neg	Neg	Neg	Neg	No adverse transfusion reaction.	Transfused in surgery.
6	3	1+	4+	1+	3+	No adverse transfusion reaction.	Use piperacillin-tazobactam for 5 days
9	1.5	1+	4+	1+	3+	**Symptoms and Signs**: Chills, fever, body temperature: 38.5°C, pulse: 95 beats/min, blood pressure (BP): 117/64mmHg. The temperature rose to 39.0°C half an hour later. **Treatment:** Stopped transfusion immediately and dexamethasone 10mg was administered. Merrill 7.5ml orally for antipyretic.	After 2 days of discontinuation, piperacillin-tazobactam were reapplied on day 1.
10	3	2+	4+	1+	3+	**Symptoms and Signs:** Fever, temperature fluctuation range 36.8°C-39°C;plus: 100 beats/min, BP 97/67mmHg, oxygen saturation 98%. **Treatment:** Closely monitor.	After 2 days of discontinuation piperacillin-tazobactam were reapplied on day 2.
14	3	2+	4+	1+	3+	**Symptoms and signs:** Chest distress; backache. **Treatment:** Intravenous infusion of dexamethasone.	3 days after changing antibiotics.
20	3*	2+	4+	1+	3+	Prophylactic use of dexamethasone, no adverse transfusion reaction.	9 days after antibiotic change, 8 days after hormone therapy, 4 days after IVIG therapy.

1. The numbers in “Days” column indicate the day after the operation, the number “0 “ indicates the day on operation.

2. The “Neg” indicates a negative reactive result; the “+” indicates a positive reactive result, and the number before “+” indicate the serologic reaction strength.

3. The “*” indicate the antigen of the transfused RBC is Jka(-) and Leb(-).

4. The medication treatment: the changed antibiotics is meropenem; the hormone therapy is prednisone; IVIG indicates Intravenous immunoglobulin.

### Transfusion-related Serological Test Results

The serological typing reagents used were commercial monoclonal IgM, IgG, and IgA from Sanquin Reagents B. V. (Netherlands). The Microcolumn gel or saline test tube method was used to type the patient’s antigen according to the manufacturer’s specifications. On the 5th day after surgery, antibody screening was positive, I(1+), II(1+), III(1+w), direct antiglobulin direct test (DAT) (3+) for all IgG, IgG+C3d, and C3d. The patient’s antigen typing results were as follows: DccEe, S-s+, Jk(a-b+), M+N+, Le(a+b-), Fy(a+b+), Wra+, Mia+, Lu(a-b+), Dia+, and P1+. Binding antibody identification results indicated that anti-Jk(a) combined with anti-Le(b) mediated the delayed hemolytic transfusion reaction (**Tables 3**–**5**).

**Table 3:** Summary of *Immunohematology* of present case.

Blood types	ABO	Rh	Duffy	Kidd	Lewis	P1	MN	Ss	Wra	Mia	Dia	Luth
Results	A	DccEe	Fy(a+b+)	Jk(a-b+)	Le(a+b-)	+	M+N+	S-s+	+	+	+	Lu(a-b+)

**Table 4:** Results of antibody screening test in the plasma and eluate.

Antibody Screening Cells	Rh-hr					Kell			Duffy			Kidd		Lewis		P	MNS				Luth	Colt	Xg	Experimental Results	
	C	D	E	c	e	K	k	Kpa	Jsa	Fya	Fyb	Jka	Jkb	Lea	Leb	P1	M	N	S	s	Lua	Cob	Xga		
																								Plasma	Eluate
I	+	+	0	0	+	0	+	0	0	+	0	+	0	0	+	+	0	+	0	+	+	0	+	1+	1+
II	+	0	+	+	0	0	+	0	nt	+	+	+	+	+	0	0	+	0	+	0	0	0	+	1+	1+
III	0	0	0	+	+	+	+	0	0	0	+	0	+	0	0	+	0	+	0	+	0	0	+	w	w

**Table 5:** Reaction results with spectrum cells of patient in plasma and eluate.

	Rh-Hr					Kell						Duffy		Kidd		Lewis		P	MNS				Luther		Xg	Experimental Results		
	C	D	E	c	e	K	k	Kpa	Kp^b^	Jsa	Jsb	Fya	Fyb	Jka	Jkb	Lea	Leb	P1	M	N	S	s	Lua	Lub	Xga	IgG+C3		
																										Plasma	Elute	Papain
1	+	+	0	0	+	0	+	0	+	/	+	+	0	+	0	0	+	+	+	0	0	+	0	+	+	1+	w	3+
2	+	+	0	0	+	+	+	0	+	0	+	0	+	0	+	0	+	+	+	+	+	0	0	+	/	w	0	3+
3	0	+	+	+	0	+	+	0	+	0	+	+	+	0	+	+	0	+	0	+	+	+	0	w	/	w	0	3+
4	0	+	0	+	+	0	+	0	+	/	+	+	0	+	0	0	+	+	+	0	+	0	+	+	+	1+	1+	3+
5	+	0	0	0	+	0	+	0	+	/	+	+	0	+	+	0	+	+	0	+	+	+	0	+	+	w	0	3+
6	0	0	+	+	0	0	+	0	+	/	+	0	+	+	+	0	+	+	+	0	+	0	0	+	+	1+	1+	3+
7	0	0	0	+	+	0	+	0	+	0	+	+	0	+	0	0	0	0	+	+	0	+	+	+	/	w	1+	3+
8	0	0	0	+	+	0	+	+	+	0	+	+	0	0	+	0	+	+	+	+	0	+	0	+	/	w	1+	3+
9	0	0	0	+	+	+	0	0	+	/	+	0	+	0	+	+	0	+	+	+	0	+	0	+	+	w	0	3+
10	0	0	0	+	+	0	+	0	+	0	+	+	+	+	0	0	+	+	0	+	+	0	0	+	/	w+	0	3+
11	+	+	+	0	+	0	+	0	+	0	+	0	+	+	+	+	0	0	+	+	0	+	0	+	/	0	0	3+
12	+	+	+	+	0	0	+	0	+	/	+	+	0	+	+	0	+	+	+	+	0	+	0	+	/	1+	w	3+
13	0	0	0	+	+	0	+	0	+	/	+	0	+	0	+	0	+	+	0	+	0	+	+	+	+	1+	1+	3+
14	+	0	0	+	+	0	+	+	+	0	+	0	+	0	+	0	+	+	+	+	0	+	0	+	0	w	0	3+
15	0	+	+	+	+	0	+	0	+	0	+	+	+	+	+	0	+	+	+	+	+	+	+	0	+	1+	1+	3+
16	+	0	0	+	+	0	+	0	+	/	+	+	+	+	+	0	+	+	+	+	0	+	0	+	/	2+	1+	3+

### Drug antibody test results

Antibody test reagents were purchased from Zhongjiwantai Company (China). The microcolumn gel method was performed according to the manufacturer’s protocol. Before the use of piperacillin, no antibody to piperacillin was detected in the plasma and RBCs of patients; when piperacillin was discontinued, antibody to piperacillin was detected in both plasma and RBCs. When discharged, only antibody to piperacillin was detected on RBCs, the titer was 16, and the titer was lower than that when piperacillin was discontinued, indicating that the amount of drug antibody in the body was significantly reduced. Twenty days after discharge, plasma and RBCs were negative for drug antibodies (**Table 6**).

**Table 6:** Results of piperacillin antibody test in the red blood cell and plasma.

Results	DAT			Patient Plasma + Piperacillin treated red blood cells	Positive Ctl + Piperacillin treated red blood cells	Negative Ctl + Piperacillin treated red blood cells	Patient Plasma + Non-Piperacillin treated red blood cells
	IgG	C3d	Ctl	I	II	III	IV
Before piperacillin treatment	0	0	0	0	2+	0	0
Stop piperacillin treatment	1+	2+	0	1+	2+	0	1+
Discharge	1+	1+	0	1+	2+	0	0
20 days after discharge	0	0	0	0	2+	0	0

### HLA typing

HLA-DRB1 and -DQB1 were detected after deoxyribonucleic acid (DNA) extraction from peripheral blood, following the protocol as previously described (6, 7). The results showed a haplotype of HLA-DQB1*03:03-DRB1-09*01, and DQB1*03:03-DRB1*14:01.

## Discussion

We have reported herein a case of a severe postoperative immune hemolytic reaction caused by the administration of piperacillin. Fortunately, we quickly recognized and identified the drug reaction to piperacillin and were able to immediately stop the administration of piperacillin. We were also able to administer the appropriate treatment, which included blood transfusions, IVIG, and glucocorticoids. This case highlights that doctors must remain vigilant about the potential adverse effects of piperacillin when administering the drug in the clinic. Furthermore, once an adverse reaction occurs, the timely discontinuation of piperacillin and appropriate drug and blood transfusion interventions are key to achieving a good outcome.

Piperacillin is the third most common antibiotic to cause serious adverse drug reactions, as previously reported, among which acute immune hemolysis reaction is one of the more serious reactions (8). Acute piperacillin-induced DIIHA is a complex process mediated primarily by immune mechanisms, which occurs through two main mechanisms: non-drug- and drug-dependent antibodies. Non-drug-dependent antibodies behave similarly to autoantibodies and can cause AIHA, while drug-dependent antibodies react with RBCs only in the presence of drugs (9). The most common mechanism by which piperacillin induces DIIHA is from drug-dependent antibodies, which occurs through three different mechanisms. In the first mechanism, piperacillin acts as a hapten, binding to erythrocyte membranes and triggering the production of high-titer anti-drug antibodies. These antibodies attach to the erythrocyte membrane, causing DAT positivity and subsequent hemolysis. The second is the non-specific absorption of piperacillin on the erythrocyte membrane, leading to membrane modification and promoting immune-mediated hemolysis. DAT is also positive in this mechanism, however, it does not always lead to hemolytic anemia. The third mechanism is attributed to the binding of the preformed immune complex of piperacillin and antibodies to the erythrocyte membrane, which activates the complement and causes severe intravascular hemolysis (10). Both IgG and C3d antibodies were present in this case, indicating persistent intravascular and extravascular hemolysis.

In our case, the patient’s Hb level dropped sharply to 42 g/dL, although they no longer declined once the piperacillin was discontinued. After the administration of piperacillin, the RBC count decreased significantly, from 2.7 to 1.8 × 1,012/L, and due to serious RBC damage, the transfusion could not inhibit the antibody-mediated hemolysis. The lowest RBC level was 1.3 × 1,012/L before the cessation of piperacillin, by which time 7.5 U (1.5 L whole blood preparation) had been transfused. As the hemoglobin decreased from 89 to 42 g/dL, approximately 3–4 L of RBCs were destroyed. Additionally, the patient’s DAT was positive, indicating the possibility of continuous RBC destruction. The detection of piperacillin drug antibodies provides the most direct evidence for clinical diagnosis and treatment. Furthermore, drug-dependent antibodies can also target platelets, resulting in thrombocytopenia (11). The binding sites of drug-dependent antibodies can be localized to GPIIb/IIIa and GPIb/IX in platelets. In our patient, the platelets decreased from 74 to 49 × 109/L (the lowest point) after surgery, but were 66 × 109/L at the time of the patient’s discharge, and 110 × 109/L at her follow-up 20 days post-discharge, indicating that in this case, piperacillin primarily resulted in the immune hemolysis of RBCs and the effect on platelets was less obvious than that of RBCs. Additionally, drug-associated trace metabolites or degradation products can also induce severe or life-threatening DIIHA, suggesting that attention must be paid to the possibility of inducing immune hemolysis when administering piperacillin. Moreover, the possibility of immune hemolysis should be monitored even after the discontinuation of piperacillin (12).

The antibody screening test, which screened positive for antibodies, was the biggest serological difference between this case and previous piperacillin-induced hemolysis. The reaction pattern between the patient’s plasma and reagent red blood cells with different antigens showed different intensity, suggesting the possible presence of irregular antibodies, which often leads to ineffective transfusions and hemolysis. The antibody identification results suggested that anti-Jk(a) combined with anti-Le(b) mediated a delayed hemolytic transfusion reaction. However, after a transfusion of red blood cells matching Jk(a) and Le(b) antigens, the anemia of the patient remained unimproved. We became aware and validate whether the hemolytic anemia was caused by drug antibodies, combined with blood transfusion inefficacy and Naranjo’s assessment scoring method. However, antibody screening has delayed the identification of drug antibodies. Although antibodies to piperacillin caused fewer cases of antibody screening positive, a previous study in a 50-year-old woman exposed to piperacillin, serological reactivity (DAT and IAT) increased significantly, and autoantibodies with Rhesus-e specificity (autoanti-e) were detectable in the patient’s plasma on day 12 of antibiotic treatment. These results suggest that irregular antibodies may delay the diagnosis of drug antibodies and that drug-induced hemolytic anemia transfusion should also consider irregular-like antibody specificity to avoid more severe hemolysis.

Class II HLA molecules, including HLA-DR, -DQ, and -DP, are expressed on the surface of antigen-presenting cells (APCs), including B cells, dendritic cells, and monocytes, presenting endogenous antigens that are recognized by T-cell receptors as part of the adaptive immune response. APCs combine with antigenic peptides to bind CD4+ T helper cells, activating B cells and subsequently producing antibodies against the corresponding epitopes (13). HLA-DRB1 and -DQB1 molecules account for > 90% of all HLA class II molecules (14). As previously reported, the HLA-DRB1-09*01-DQB1*03:03 loci and linked haplotypes are prone to produce isoimmune anti-E antibodies in the Chinese population (15, 16). In a prior study, the HLA typing of a patient with ceftriaxone-induced hemolytic anemia showed DRB1*04:05 and DQB1*04:01 haplotypes, which are more common among patients with autoimmune hepatitis (16–19), and anti-Fya-mediated delayed hemolytic transfusion reactions related to HLA-DRB1*04:03 (20), suggesting that people carrying specific HLA risk alleles may be prone to producing drug-mediated antibodies. In the future, special attention should be paid to the hemolytic response of drug antibodies in patients with these HLA alleles.

The immune hemolysis response has immune memory; therefore, initial exposure to piperacillin stimulates the immune system to produce antibodies against the drug or its complex with RBCs. Once antibodies are produced, the memory B cells remain in the body (21), allowing a quicker and increased production of antibodies against the drug-RBC complex when exposed to the same drug again (18). This accelerated immune response, however, can lead to a more rapid and severe hemolytic reaction. A history of past development of antibodies to related drugs, and consideration of alternative antibiotics where appropriate, is a key step in controlling the risk of developing severe immune hemolysis with the repeat administration of piperacillin in the future (3). Our titer of the drug-dependent antibodies was 16, with patients (8, 22) showing titer of 2, 128, and 256 with piperacillin-induced severe immune hemolytic anemia. Low antibody titer can also induce severe drug-induced hemolysis by piperacillin. After the development of piperacillin drug antibodies, treatment with IVIG and hormone therapy may be necessary to alleviate further hemolysis; however, the specific role of IVIG in this success is unclear. After the patient was identified as having an antibody reaction, she successfully recovered and was discharged from the hospital through a combination of drug cessation, blood transfusion, IVIG, corticosteroids, oxygen inhalation, and close vital sign monitoring.

## Conclusion

Piperacillin administration may trigger a severe and potentially fatal hemolytic reaction in some patients, and HLA-DQB1*03-DRB1-09*01 may be a susceptible gene target to the induction of piperacillin antibodies. Effective treatment includes blood transfusion, IVIG therapy, corticosteroids, and related vital sign monitoring. In patients with positive antibody screening in particular, antibody information should be followed up after the cessation of piperacillin to avoid delayed hemolytic transfusion reactions mediated by alloimmunity in the future.

## Data Availability

The raw data supporting the conclusions of this article will be made available by the authors, without undue reservation.

## References

[1] Zanetti RC Biswas AK . Hemolytic anemia as a result of piperacillin/tazobactam administration: a case report and discussion of pathophysiology. Mil Med. (2013) 178:e1045–7. doi: 10.7205/MILMED-D-12-00512 24005557

[2] Hill QA Stamps R Massey E Grainger JD Provan D Hill A . Guidelines on the management of drug-induced immune and secondary autoimmune, haemolytic anaemia. Br J Haematol. (2017) 177:208–20. doi: 10.1111/bjh.2017.177.issue-2 28369704

[3] Loriamini M Cserti-Gazdewich C Branch DR . Autoimmune hemolytic anemias: classifications, pathophysiology, diagnoses and management. Int J Mol Sci. (2024) 25:4296. doi: 10.3390/ijms25084296 38673882 PMC11049952

[4] Mulder FVM Evers D de Haas M Cruijsen MJ Bernelot Moens SJ Barcellini W . Severe autoimmune hemolytic anemia; epidemiology, clinical management, outcomes and knowledge gaps. Front Immunol. (2023) 14:1228142. doi: 10.3389/fimmu.2023.1228142 37795092 PMC10545865

[5] Mayer B Bartolmas T Yurek S Salama A . Variability of findings in drug-induced immune haemolytic anaemia: experience over 20 years in a single centre. Transfus Med Hemother. (2015) 42:333–9. doi: 10.1159/000440673 PMC4678312 26696803

[6] Ou G Xu H Yu H Liu X Yang L Ji X . The roles of HLA-DQB1 gene polymorphisms in hepatitis B virus infection. J Transl Med. (2018) 16:362. doi: 10.1186/s12967-018-1716-z 30563535 PMC6299522

[7] Ou GJ Ji X Wang J Liu Z . Identification of the novel allele, HLA-DRB1*09:30, by sequence-based high resolution typing. HLA. (2017) 90:379–80. doi: 10.1111/tan.2017.90.issue-6 28980777

[8] Wu Y Wu Y Guo G Zeng J Liu Y Wu Y . Piperacillin-tazobactam induced immune hemolytic anemia led to increased renal impairment and eventual death from multiple organ failure in a patient with hypertensive nephropathy: case report and literature review. BMC Nephrol. (2023) 24:173. doi: 10.1186/s12882-023-03235-w 37316798 PMC10268429

[9] Arndt PA . Drug-induced immune hemolytic anemia: the last 30 years of changes. Immunohematology. (2014) 30:44–54. doi: 10.21307/immunohematology-2019-098 25247622

[10] Pierce A Nester T . Education Committee of the Academy of Clinical Laboratory, and Scientists, Pathology consultation on drug-induced hemolytic anemia. Am J Clin Pathol. (2011) 136:7–12. doi: 10.1309/AJCPBVLJZH6W6RQM 21685026

[11] Sathiasekar AC Deepthi DA Sathia Sekar GS . Drug-induced thrombocytopenic purpura. J Pharm Bioallied Sci. (2015) 7:S827–9. doi: 10.4103/0975-7406.163595 PMC4606724 26538982

[12] Garratty G . Immune hemolytic anemia caused by drugs. Expert Opin Drug Saf. (2012) 11:635–42. doi: 10.1517/14740338.2012.678832 22502777

[13] Roehmel J Specht P Staab D Schwarz C Salama A Mayer B . Risk of piperacillin-induced hemolytic anemia in patients with cystic fibrosis and antipseudomonal treatment: a prospective observational study. Transfusion. (2019) 59:3746–54. doi: 10.1111/trf.v59.12 31724753

[14] Gassner C . Responder individuality in red blood cell alloimmunization. Transfus Med Hemother. (2014) 41:403–4. doi: 10.1159/000369599 PMC4280444 25670927

[15] Gouw JW Jo J Meulenbroek L Heijjer TS Kremer E Sandalova E . Identification of peptides with tolerogenic potential in a hydrolysed whey-based infant formula. Clin Exp Allergy. (2018) 48:1345–53. doi: 10.1111/cea.2018.48.issue-10 29974988

[16] Shang W Ou G Ji X Chen J Wang J Jiang Y . Investigating the correlation between HLA-II gene polymorphism and rhE alloimmunization in pregnant Chinese women. Indian J Hematol Blood Transfus. (2023) 39:662–9. doi: 10.1007/s12288-023-01632-7 PMC10542046 37786831

[17] Tian L Hou L Wang L Xu H Xiao J Ying B . HLA-DRB1*09:01 allele is associated with anti-E immunization in a Chinese population. Transfusion. (2018) 58:1536–9. doi: 10.1111/trf.2018.58.issue-6 29516495

[18] Kong Y Xiao J Tian L Xu Y . The influence of HLA allele and haplotype on RhE alloimmunization among pregnant females in the Chinese Han population. Vox Sanguinis. (2024) 119:737–44. doi: 10.1111/vox.v119.7 38637118

[19] Lou C Liu M Ma T Yang L Long D Li J . Case report: Decreased hemoglobin and multiple organ failure caused by ceftizoxime-induced immune hemolytic anemia in a Chinese patient with Malignant rectal cancer. Front Immunol. (2024) 15:1390082. doi: 10.3389/fimmu.2024.1390082 38756782 PMC11096485

[20] Matsuno T Matsuura H Fujii S Suzuki R Sugiura Y Miura Y . Anti-Fy(a)-mediated delayed hemolytic transfusion reaction following emergency-release red blood cell transfusion: possible involvement of HLA-DRB1*04:03 in the Japanese population. Int J Hematol. (2022) 115:440–5. doi: 10.1007/s12185-021-03242-3 34714525

[21] Cyster JG Wilson PC . Antibody modulation of B cell responses-Incorporating positive and negative feedback. Immunity. (2024) 57:1466–81. doi: 10.1016/j.immuni.2024.06.009 PMC11257158 38986442

[22] Mayer B Yurek S Salama A . Piperacillin-induced immune hemolysis: new cases and a concise review of the literature. Transfusion. (2010) 50:1135–8. doi: 10.1111/j.1537-2995.2009.02544.x 20051057

